# Denialism: repudiation of anti-Indigenous racism in healthcare in Canada

**DOI:** 10.3389/fpubh.2026.1766047

**Published:** 2026-03-03

**Authors:** Mariam Abdelmalek, Patricia Farrugia

**Affiliations:** 1 Faculty of Health Sciences, McMaster University, Hamilton, ON, Canada; 2 Division of Orthopedic Surgery, Department of Surgery, McMaster University, Hamilton, ON, Canada

**Keywords:** Indigenous, First Nations, racism, discrimination, healthcare

## Abstract

Historically, healthcare in Canada has functioned as a tool of colonialism negatively impacting Indigenous peoples, producing endured health inequities and continues to shape contemporary health pathways for Indigenous patients, families and communities. Indigenous patients’ healthcare experiences are shaped by encounters with anti-Indigenous racism, which directly affect both the quality of care they receive and their overall well-being. Anti-Indigenous racism in healthcare is sustained by denialism. Denialism is process through which anti-Indigenous racism is minimized, rationalized, or acknowledged without meaningful action, allowing colonial harms and inequities to persist. Using a framework that examines the intersection between denialism and anti-Indigenous racism across systemic, interpersonal, and societal levels, this perspective article analyzes how denialism translates recognition of inequity into inaction, stereotypes, and bias; thereby sustaining anti-Indigenous racism in healthcare settings. Addressing denialism is essential to dismantling structural racism in healthcare and to advancing meaningful, accountable change for Indigenous peoples in Canada.

## 1 Introduction

In How to Be an Antiracist, Ibram X. Kendi writes, “Racism is one of the fastest spreading and most fatal cancers humanity has ever known” (1). Like cancer, racism mutates and adapts to survive, often shifting in form, becoming harder to detect, and embedding itself deeper into the social life. For Indigenous^1^ peoples in Canada, racism has always been at the core of colonial advancement practices (2). Despite the public acknowledgment of this harm, colonialism continues to shape their daily realities, especially within healthcare systems (3).

Historically, healthcare in Canada has been weaponized as a tool of colonialism (3). Indigenous communities were subjected to deliberate medical neglect and abuse: fomented smallpox epidemics, avoidable tuberculosis deaths, forced sterilizations, medical experimentation in residential schools and Indian hospitals, and the systemic removal of children from their communities (4). These atrocities were not anomalies but part of a larger effort to erase Indigenous identity and autonomy. While the Canadian government formally recognized the harms of colonial policies; most notably in the 2015 Truth and Reconciliation Commission (TRC) report, this recognition did not dismantle the structures that allowed racism to persist (5). Instead, racism has assumed more insidious forms, embedded in stereotypes, systemic neglect, and societal narratives that continue to shape Indigenous experiences today (6, 7).

Colonialism has profoundly shaped how Indigenous peoples are perceived in Canadian society, fostering persistent stereotypes and false narratives that continue to represent them through a negative colonial lens. These misrepresentations are not relics of the past; they remain deeply embedded in societal structures and are at the root of many contemporary inequities. Despite official recognition of historical injustices, beginning with Prime Minister Stephen Harper’s 2008 formal apology for the harms caused by the residential school system, and followed by the Truth and Reconciliation Commission of Canada’s Final Report in 2015, the legacy of colonialism continues to manifest through various forms of racism (2, 3, 8).

These expressions of racism contribute to ongoing disparities that disproportionately affect Indigenous communities, including poorer physical and mental health outcomes, lower socioeconomic status, higher incarceration rates, and overall diminished well-being. The persistent impact of colonialism and deep-rooted socioeconomic inequities has created a context for Indigenous peoples that is distinct from other marginalized groups (9). Therefore, comparisons with other racialized communities in Canada can be misleading, as the Indigenous experience is uniquely shaped by colonization, displacement, and historical trauma. Without continued validation and recognition of historical events and their impact on the current and future health of Indigenous people in Canada, reconciliation efforts to address anti-Indigenous racism will be futile.

## 2 The history of racism against Indigenous peoples in the Canadian healthcare

Indigenous peoples represent approximately 5% of the population in Canada, comprised of almost 2 million people (10). Each Indigenous community is unique with cultural practices, ceremonies, and languages; as well as historical and colonialist encounters which have resulted in continued health inequities. Indigenous populations continue to face disproportionately high rates of chronic disease, mental health challenges, and reduced life expectancy (11). With ideals of assimilation from Euro Christian settlers, supported by stereotypical beliefs of “savages” and governmental politically driven interference, fashioned the health and well-being challenges we see today for Indigenous people in Canada (41). These disparities are not simply the result of personal choices or lack of access to care but are rooted in longstanding colonial structures, systemic racism, and intergenerational trauma, legacies of policies like the Indian Act and the residential school system (11).

Colonialism in Canada is not a closed chapter of history but an ongoing structure that continues to shape healthcare by suppressing Indigenous knowledge, erasing lived experiences, and normalizing inequity (3). The disruption of Indigenous culture and identity continues to influence health outcomes negatively. The social determinants of health, such as income, housing, education, and access to culturally safe services, are significantly different for Indigenous peoples compared to the broader Canadian population, reflecting ongoing colonial impacts (11). While the Canadian healthcare system strives for neutrality and universality, it sometimes faces challenges in fully addressing the unique needs and historical contexts of Indigenous communities, which can contribute to ongoing gaps in care and outcomes (7).

Indigenous worldviews on health differ profoundly from Western medical models: Indigenous understandings of well-being often emphasize holistic, interconnected dimensions of life. For example, the Anishinabek concept of *mno bmaadis*, translated as “living the good life” or “being alive well,” reflects a broader notion of health that goes beyond physical wellness to include spiritual, emotional, and community dimensions (7). In a recent study of traditional medicine and Indigenous perspectives on health, the dismissal by Western health care providers in valuing traditional knowledge as a part of a patients’ health care of journey created disappointment in patients (12). The urban Indigenous health clinic where many participants were accessing health care services, were also seen as “lesser than” Western health care services (12). Disregarding these worldviews not only leads to cultural erasure but actively undermines the effectiveness and trustworthiness of healthcare marginalizing Indigenous knowledge systems, failing to provide culturally safe services, and reinforcing the health disparities.

Ongoing inattention to effectively address the persistent health care inequities for Indigenous people in Canada contributes to a cycle of colonial-based values and action toward the well-being for Indigenous people. Colonialist ideals, present since the Euro Christian settlers arrived, continue to define and guide health care priorities for the government (41). As these ideals were rooted in Western superiority, they continue to devalue Indigenous sovereignty and ways of knowing for health in many ways through inferiority and anti-Indigenous racism. By dismissing the truth and impact of colonial driven historical events in Canada for Indigenous people, the result is denialism. Denialism of the current state of health for Indigenous people in Canada purpurates the systems and structures of settler colonialism.

This paper focuses on a critical but underexplored dimension of anti-Indigenous racism in healthcare: *denialism*. Denialism refers to the refusal to acknowledge, or the deliberate minimization of the historical and ongoing harms experienced by Indigenous peoples (13). It manifests through the dismissal of Indigenous accounts of mistreatment, the resistance to policy changes that address racism, and the collective societal tendency to frame colonialism as a closed chapter of history. This narrative review addresses this critical gap by exploring the research question: *What is denialism in the context of anti-Indigenous racism in Canadian healthcare systems, and how does it act as a systemic barrier to achieving health equity?*

## 3 What is denialism and its connection to anti-Indigenous racism in healthcare?

Denialism is a structural and interpersonal process through which institutions and individuals minimize, rationalize, or acknowledge but fail to act upon racism, thereby sustaining racial inequities while presenting inaction as neutrality, inevitability, or goodwill.

In How to Be an Antiracist, Ibram X. Kendi does not explicitly define the term denialism as a form of racism; however, he outlines the concept by describing individuals who advance racist ideas and policies yet deny their existence or refuse to act against them as inherently racist (1). Denialism, in this sense, functions both as an evasion of moral responsibility and a justification for inaction. Scholars have described denialism as a “mixture of denial and neglect” (14).

Denial involves the refusal to recognize the presence or consequences of racism, often through silence, minimization, or rationalization, while neglect refers to the failure, whether deliberate or passive, to take necessary actions to correct racial inequities (14). These two mechanisms converge to form denialism: a structural condition wherein institutions acknowledge racial disparities without committing to meaningful change (14). In book written by Stanley Cohen two forms of denialism have been identified: literal denial, or the outright rejection of facts, and implicatory denial, the acknowledgment of racism without taking action to address it (15). Other authors identify another subtler form of denialism referred to as “soft” denialism which reframes historical atrocities as “well-intentioned mistakes,” for example, the claim that residential schools were intended for education rather than assimilation and cultural erasure (16, 17). Denialism, therefore, is not simply passive ignorance but an active process that upholds systemic oppression under the guise of neutrality or goodwill.

Denialism in healthcare can be recognized through several observable institutional and clinical patterns. These include minimizing the role of racism in shaping health disparities, often attributing poor outcomes to individual behavior or cultural traits rather than to systemic injustices (18). This framing deflects attention from the long-term effects of colonization, racism, and implicit biases among healthcare professionals, all of which contribute to the lower quality of care received by Indigenous peoples in Canada (18). Denialism also manifests through acknowledgment without accountability, whereby reports and data documenting racism are formally accepted but fail to result in enforceable policy changes or meaningful shifts in clinical practice. A 2025 policy and equity analysis of systemic racism in Canadian healthcare describes a “recurring pattern in which the recommended action is not taken following a review, suggesting organizational resistance to change,” even after reports documenting racism and harm to Indigenous and Black patients (19). Additionally, institutions may deflect responsibility through claims of neutrality, asserting that healthcare systems are purely merit-based despite persistent and well-documented disparities in outcomes. A 2023 paper in the Journal of Medical Ethics argues against a “color blind” or race-neutral stance, pointing to extensive evidence that racial and ethnic minority patients receive suboptimal care and experience worse outcomes, thereby challenging claims that healthcare is purely merit-based or race-blind (20). As a result, denialism implicitly undermines efforts to achieve health equity. When healthcare providers and policymakers deny or downplay the impact of systemic racism, they fail to confront its root causes and instead implement superficial or ineffective solutions.

Denialism about the existence of racism can lead to neglect for many marginalized communities in health care settings. Neglect can occur with intentional or unintentional disregard of people in situations which may be uncomfortable for some care providers, or the result of avoidance of negative effects that may be presence in a care situation. Denialism may discount the contribution of racism in an Indigenous patients’ health care experience, and neglect in an Indigenous patient’s health care experience enables anti-Indigenous racism to continue. This paradox is evident in the tragic case of Brian Sinclair (21).

In 2008, Brian Sinclair, a 45-yr old Indigenous male sought out care at an emergency room at Winnipeg Health Sciences Center in Winnipeg, Manitoba, Canada. After being registered at the nursing triage station, he was ignored for 34 h by all levels of healthcare staff, in the ER department’s waiting room, despite demonstrating physical signs and symptoms of infection. He was found deceased in his wheelchair, unattended by a hospital security guard. As his case was reviewed at the institutional and provincial level, several healthcare providers admitted that harmful Indigenous stereotypes contributed to the neglectful care of Mr. Sinclair (21). Neglect in healthcare continues to exist reinforcing health inequities, preserving anti-Indigenous racism, and sustaining denialism for health status of Indigenous peoples in Canada.

While denialism intersects with related concepts such as claims of neutrality, institutional inertia, and epistemic injustice, it is not reducible to any one of these. Denialism, as used in this paper, refers to a persistent state of mind within individuals and institutions that minimizes, denies, or rationalizes the needs and rights of Indigenous peoples, and thereby normalizes the neglect of their health and well-being. Claims of neutrality describe how institutions present themselves as objective or “race-blind,” often insisting that policies are merit-based even when inequitable outcomes are evident (20). Institutional inertia refers to the tendency of systems to resist or delay change, resulting in policy inaction or superficial reform even when problems are acknowledged (22). Epistemic injustice captures how the knowledge and experiences of Indigenous peoples are routinely discredited, devalued, or rendered invisible within healthcare decision-making (23). In this sense, neutrality claims, institutional inertia, and epistemic injustice are manifestations and mechanisms through which a denialist mindset appears and operates. What denialism uniquely names is this ongoing, justificatory work that converts knowledge of harm into inaction, thereby transforming neutrality and inertia from background features into mechanisms that sustain anti-Indigenous racism.

## 4 Intersectionality of types of denialism: how it manifests into anti-indigenous racism in healthcare

**Figure 1:**
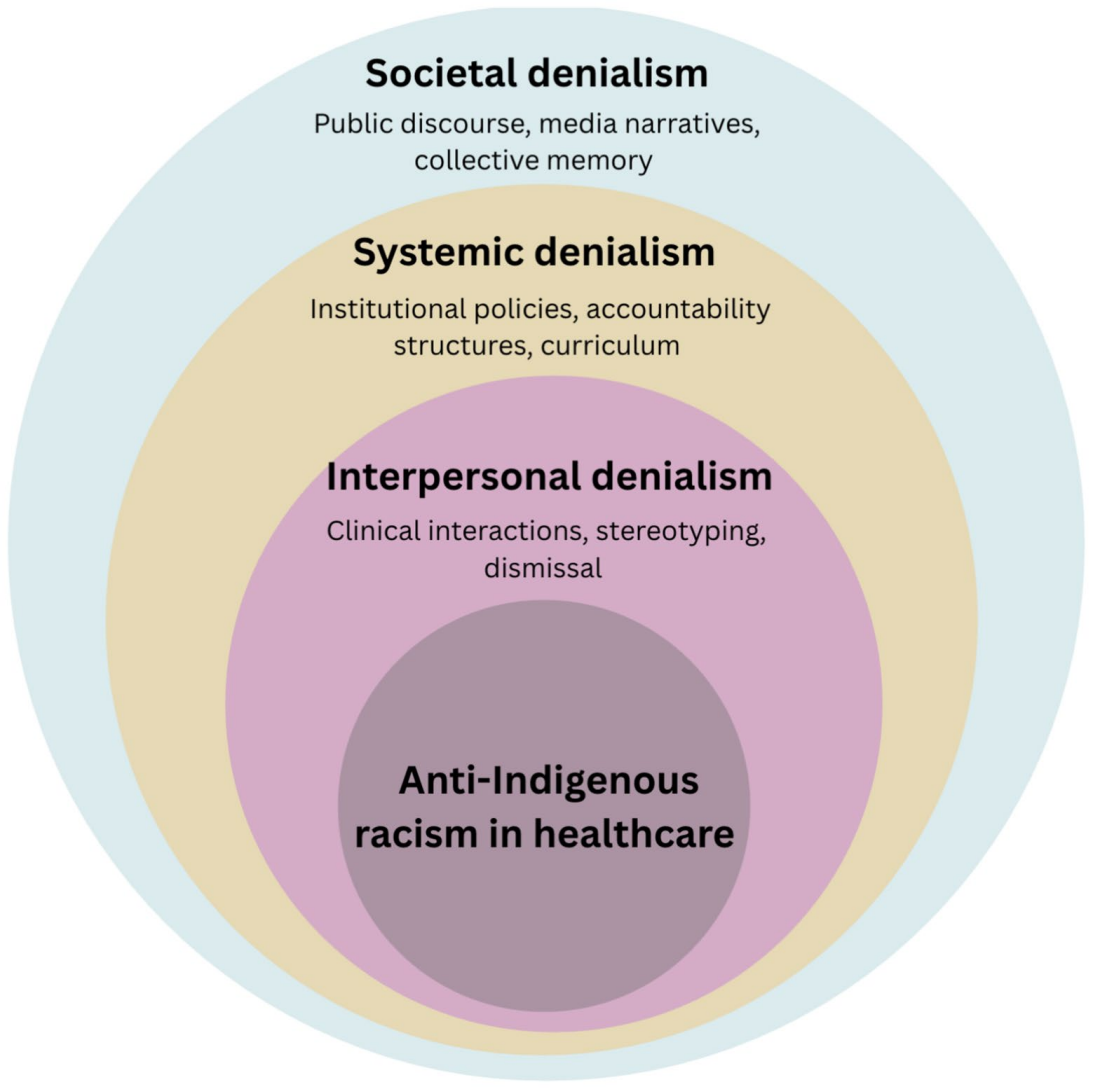
Levels of denialism shaping anti-Indigenous racism in healthcare.

Denialism in healthcare can exist on several levels for Indigenous peoples in Canada. Several levels including interpersonal, systemic, and societal denialism reinforces socioeconomic disparities, normalizes systemic racism and manifests in policy inaction by healthcare institutions and government. Through a literature review of existing research, critical pathways and gaps exist to identify methods to define three interconnected forms of denialism: systemic, interpersonal, and societal denialism (Figure 1) systemic denialism occurs when institutional policies and practices fail to recognize or respond to the distinct needs of Indigenous peoples. Interpersonal denialism occurs in situations where healthcare providers discount or trivialize Indigenous patients’ experiences, further eroding trust in the system. Societal denialism occurs in broader Canadian society resists acknowledging the enduring impacts of colonialism and racism in Indigenous health.

### 4.1 Systemic denialism

Systemic denialism in healthcare refers to institutional policies and practices that fail to acknowledge or appropriately respond to the specific needs and experiences of Indigenous peoples. One example of systemic denialism is the privileging of Western biomedical knowledge over Indigenous healing practices (24). This leads to the marginalization of Indigenous healers and the exclusion of their customs from healthcare decision-making. For example, requests to build culturally significant spaces such as sweat lodges in healthcare facilities serving Indigenous populations are often deprioritized in favor of technological upgrades (24). These practices influence what is recognized as legitimate medical knowledge, how resources are distributed, which patients receive care, and the quality of that care. Such exclusion reflects a denial of Indigenous cultural needs and neglect of their relevance in achieving health equity. The In Plain Sight report on Indigenous-specific racism and discrimination in B. C. Health Care powerfully illustrates how systemic denialism impacts the training of healthcare professionals (3). A student shared:

*“I was told by a faculty member to ‘leave your Indianness at the door.’ As a graduate student, I was told that my offer to mentor Indigenous students new to nursing was ‘not possible.’ I was labelled as ‘one-dimensional’ and ‘too Indian’ for advancing issues of importance to Indigenous people while in nursing leadership positions. Continuous roadblocks and excuses were the norm in response to any ideas related to integration of Indigenous perspectives in both my practice and my nursing leadership roles”* (3).

Tokenistic measures are another example of systemic denialism. Tokenism refers to symbolic gestures that create the appearance of racial equity without addressing or challenging the root causes of systemic racism (25). In Indigenous healthcare, and more broadly, this often takes the form of superficial actions that do not result in meaningful structural change (25). A poignant example of tokenism in practice is seen in the case of Connor Sutton, a 23-year-old member of the T’Sou-ke Nation and Canadian Armed Forces, whose experience is documented in In Plain Sight (3). After seeking emergency medical help for both physical and mental health symptoms, Mr. Sutton was misdiagnosed, denied admission, forcibly restrained, and eventually confined in a psychiatric unit without clear justification or communication with his family. Despite the presence of an Indigenous liaison, the Sutton family reported that this individual had no real power to influence decisions or advocate effectively on their behalf. This illustrates the tokenistic deployment of Indigenous liaisons, offered as a symbol of culturally safe care, yet structurally disempowered to enact change. While the hospital could point to the liaison role as evidence of inclusivity, the lack of transparency, accountability, and culturally appropriate care reflects the unchanged racist structure of the healthcare system. The Sutton family’s experience shows how tokenism can mask systemic racism, offering the illusion of progress while reinforcing institutional neglect and harm (3). The tokenistic deployment of Indigenous liaisons acts as an elaborate act of systemic denialism, allowing the institution to point to a superficial solution while neglecting and denying the profound, acknowledged need for genuine structural reform.

### 4.2 Interpersonal denialism

Interpersonal denialism occurs when healthcare providers discount, trivialize, or outright ignore the experiences and needs of Indigenous patients, further eroding trust in the healthcare system. Unlike systemic denialism, which is embedded in institutional policies, interpersonal denialism manifests in direct interactions, often through deeply rooted stereotypes and prejudices that shape the attitudes and behaviors of healthcare workers.

Indigenous patients are frequently subjected to harmful assumptions that portray them as less deserving of care (3). Common stereotypes include:

- **Alcoholics or drug-seeking**: Patients may be presumed to be under the influence or experiencing substance-related issues, which can lead healthcare staff to dismiss or misinterpret their symptoms. An example of this harmful assumption is the death of Brian Sinclair, a First Nations man who was ignored in an emergency department which could have been due to staff incorrectly attributing his condition to substance use. Such stereotypes, particularly assumptions of increased alcohol use, can shape clinical judgment and result in delayed or inadequate care. Furthermore, patients requesting pain relief are assumed to have ulterior motives, such as obtaining medication for misuse or resale (3).
- **Irresponsible or non-compliant to medical care**: Patients are blamed for not taking responsibility for their health or failing to follow aftercare instructions (3). Healthcare providers may not be aware of the socioeconomic and geographical barriers to accessing regular health care caused by colonialism, impacting Indigenous peoples (42).
- **Belief that Indigenous individuals are unfairly advantaged**: Some healthcare workers believe Indigenous patients receive preferential treatment, leading to resentment and further discrimination (3). This perception reflects broader public attitudes in Canada, where just over one in 10 respondents express explicitly negative first impressions of Indigenous peoples, often framing them as receiving “special treatment,” tax breaks, or government handouts, or as being unwilling to work, narratives that reinforce stereotypes of undeserved privilege and dependency (“Canadian Public Opinion on Aboriginal Peoples” 2016).

The impact of interpersonal denialism is especially severe for Indigenous women, who experience a unique intersection of racism and sexism in healthcare settings (3). Misogynistic stereotypes can frame Indigenous women as “squaws,” with media depicting them as sexually promiscuous casting doubt on their parenting abilities. These stereotypes lead to the devaluation of Indigenous women’s bodies and contribute to feelings of fear and unsafety in healthcare environments (3). Data highlights the scale of this issue: Indigenous men are 83% more likely than Indigenous women to report feeling “completely safe” when visiting the emergency department, with similarly stark disparities in hospital admissions (75% more likely). In British Columbia’s only women’s hospital, First Nations women left the hospital against medical advice in 2017–18 at a rate 11 times higher than non-Indigenous women (3). These disparities reflect how Indigenous women are disproportionately affected by poor health outcomes, not only compared to non-Indigenous women but also to Indigenous men.

A particularly disturbing account illustrates the violence of such mistreatment. A healthcare witness described an incident involving an Indigenous woman with a history of trauma and sexual assault. While undergoing a C-section at a B. C. hospital, she was manhandled and yelled at by an anesthesiologist. Afterward, the same physician reportedly stated, “People like her should be sterilized.” This horrific comment reflects both the enduring legacy of forced sterilizations and the ongoing disregard for Indigenous women’s dignity and humanity in healthcare settings (3).

### 4.3 Societal denialism

Societal denialism refers to the widespread reluctance within Canadian society to acknowledge the ongoing effects of colonialism and systemic racism on Indigenous health outcomes. This form of denial operates beyond the interpersonal level and is embedded in public narratives, policy discourses, and institutional practices that minimize or erase the structural roots of Indigenous health disparities.

A notable historical instance of societal denialism can be seen in the ongoing dismissal of the long-lasting impacts of colonial practices on the health of Indigenous populations today, especially residential schools (18, 26). A significant consequence of residential schools is the nutritional deprivation experienced by Indigenous children, which continues to affect their well-being in contemporary society (18). Although precise dietary quantification is difficult, survivor testimonies consistently describe diets with insufficient calories, minimal protein and fat, and a near-total absence of fresh produce (18). Children were often exposed to spoiled or contaminated food, resulting in frequent food-borne illnesses (18). The long-term biological consequences of chronic undernutrition are profound. Studies from similar high-poverty contexts and historical famines show that early-life caloric restriction leads to stunting, which alters metabolic and endocrine function contributing to obesity and increased risk of type 2 diabetes (18). Among women, these changes are linked to higher risks of preterm birth, stillbirth, and neonatal complications (18). These physiological impacts extend beyond the individual lifespan, affecting future generations: infants born to women who experienced early-life undernutrition are more likely to suffer intrauterine growth restriction and metabolic dysfunctions. Similar patterns are seen in the adult grandchildren of famine survivors, underscoring the lasting, transgenerational impact of historical trauma on Indigenous health (18).

Mainstream public discourse and even many clinical frameworks often attribute health issues such as obesity and diabetes solely to individual lifestyle choices, failing to consider the broader historical and structural factors that shape health outcomes. However, as this evidence shows, the legacy of colonialism continues to shape the health of Indigenous populations today (18). Recognizing this link challenges dominant narratives of personal responsibility and compels a re-examination of how both physicians and society at large understand Indigenous health. For physicians, this necessitates a shift toward trauma- and context-informed care that acknowledges the role of colonial history in current disparities. More broadly, it calls on Canadian society to abandon narratives that deny or neglect the enduring effects of colonialism and instead commit to understanding how the past continues to shape present and future health trajectories for Indigenous peoples.

## 5 The death of Joyce Echaquan: a case study in denialism

### 5.1 Overview of the case

The death of Joyce Echaquan on September 28, 2020, at the Center Hospitalier de Lanaudière in Joliette, Quebec, presents a profound and tragic event within the healthcare system. A 37-year-old Atikamekw woman and mother of seven, Echaquan was subjected not only to substandard medical treatment but also to overt racial abuse, institutional neglect, and a denial of culturally safe care. This incident provides evidence of the presence of the three forms of denialism discussed in the Canadian healthcare system (27).

### 5.2 Interpersonal denialism

At the individual level, Joyce Echaquan was dismissed by healthcare staff as drug-seeking and manipulative, a prejudicial stereotype that shaped the course of her treatment. Despite presenting with severe symptoms, including stomach pain and difficulty breathing, she was labeled a narcotics addict without clinical verification (27). Nurses and attendants mocked her in a video she recorded shortly before her death, uttering degrading remarks such as, “You’re only good for sex” (27). These comments exemplify interpersonal denialism, where caregivers denied her credibility, personhood, and right to compassionate care based on racialized assumptions. Notably, during the investigation following her death, the staff involved denied harboring any racist bias, asserting they would have acted similarly toward “a woman on welfare with lots of children” (27). This denial of racial prejudice, despite explicit evidence of abuse, illustrates how racism is often obscured under the guise of neutrality.

### 5.3 Institutional denialism

Institutionally, the denial of responsibility permeated both managerial and departmental responses. Staff members and leaders failed to acknowledge the gravity of the video recorded by Echaquan, and the Aboriginal liaison officer was denied access to the emergency room when Joyce Echaquan was admitted (27). Despite knowing about the recording and receiving multiple reports of mistreatment, hospital leadership failed to initiate an immediate investigation or meaningful intervention while Joyce was still alive (27). The absence of punitive or corrective actions following previous reports of derogatory comments toward other racialized patients, including a Syrian family, further highlights the culture of institutional denialism (27). Here, denial takes the form of bureaucratic inertia, willful ignorance, and failure to recognize patterns of harm that are systemic rather than incidental.

### 5.4 Societal denialism

The case of Joyce Echaquan tragically illustrates the pervasive denialism present in Canadian society when confronted with systemic racism. This denialism was evident in the immediate public reaction to Echaquan’s video (27). Despite the clear evidence of the racist abuse she suffered before her death, many individuals dismissed her experience or refused to support her, often deflecting blame or questioning her narrative instead of addressing the reality of anti-Indigenous racism in healthcare settings (27, 28). This pattern of denial persisted in the institutional response following her death, particularly concerning anti-racism training for hospital staff. Instead of viewing the training as an opportunity for growth and reflection, many participants regarded it as irrelevant or ineffective (27). Their preference for “ultra-targeted, concise” training modules indicates a resistance to engaging with the complexity of racism and a desire for superficial solutions to deep-rooted issues (27). As noted by the coroner, this attitude stems from a paternalistic mindset, which reduces racism to isolated incidents rather than recognizing it as a systemic and cultural issue embedded within institutions (27). Such dismissal not only undermines the potential for meaningful change but also perpetuates a status quo where discriminatory practices go unchallenged, minimized, or denied.

Finally, Joyce Echaquan’s death is not an isolated incident but a manifestation of intersecting forms of denialism that continue to undermine Indigenous people’s access to equitable and dignified healthcare. From the refusal to acknowledge racial bias at the interpersonal level, to the systemic failures of accountability and the suppression of Indigenous voices in medical settings, denialism operates as both a cause and a symptom of structural racism. Joyce’s Principle, developed in response to this tragedy, affirming the right of Indigenous peoples to culturally safe care and challenging institutions to confront the denial that sustains injustice. Until such denial is dismantled, the goal of health equity remains unattainable.

## 6 Discussion

Identifying the concept of denialism in an anti-Indigenous racism context demonstrates how denialism functions as a central mechanism for the normalization and reproduction of dangerous stereotypes and subsequent healthcare experiences for Indigenous populations. Denialist beliefs are more common among men, conservatives, those with anti-Indigenous attitudes, and white Canadians who strongly identify with their racial in-group (29). Building upon interpersonal racial beliefs regarding Indigenous peoples, institutional and systemic anti-Indigenous racism is then bolstered into many facets of colonial expression against Indigenous peoples. The intersection of these three avenues for denialism, are evident in the lack of movement toward Indigenous reconciliation in healthcare and beyond.

Healthcare workers, healthcare, and academic institutions when confronted with concerns of anti-Indigenous racism in a patient care experience may respond with ignorance of the impact of colonialism (39). Statement such as “we take these things seriously” can equate to the assumption of a claim of racism by an Indigenous person is exaggerated or is related to the past not the present and does not depend on other factors impacting a person’s socioeconomic status irrelevant to colonial impacts. Despite the growing body of research demonstrating the ways in which Indigenous people are often mistreated, healthcare administrators and frontline staff often deny that racism in healthcare is a determinant Indigenous peoples’ health (30). These responses obscure the impact of ongoing societal inequalities and undermine the lived experiences of Indigenous people.

Denialism is an active ideological process, influenced by common myths and beliefs in settler colonialism. According to Didier Fassin, denialism is “an ideological position whereby one systemically reacts by refusing reality and truth (31). By identifying settler colonialism as a structure rather than an event, denialism is a vital tool to ensure the current state of persistent benefits impacting settlers remain, and the colonized population, Indigenous peoples in Canada continued to suffer with muffled attestation. It is crucial to legitimize the impact of colonialism on the well-being of Indigenous peoples—through affirmation of testimony of anti-Indigenous racism, historical events and lived experiences. This can be a challenging task as the fuel of the fire against reconciliation is denialism—starting with a shift in the truth of history and those who created the socioeconomic and intergenerational trauma for Indigenous populations still in existence.

Residential school denialism does not deny the existence of the school system, but rather downplays, excuses, or misrepresents facts about the harm caused by it (32, 38). Rationalizing the existence of the Residential School System, the true intentions of many celebrated political influencers in Canada has been exposed in recent years. Egerton Ryerson, an early educational reformer advocated for the creation of religious run “industrial” boarding schools to instill “civilization” in the “North American Indian,” (33).

Elected officials in the provincial governments in Canada used social media sites to falsify the number of grave of children discovered at former residential school sites (34). A recent study examining the prevalence of residential school denialism found that nearly one in five non-Indigenous Canadians agree with denialist claims, while an equal share feel they do not know enough to offer an opinion (29). Through denialism of these events, these positions continue to circulate widely and persist ensuring the existence of anti-Indigenous racism.

Traditional medicine and Indigenous perspectives on health involve more than just disease management. It is the connection between physical, spiritual, and community for each Indigenous individual and family (37). The view is recognized and supported by the United Nations Declaration on the Rights of the Indigenous peoples upholds the rights of Indigenous peoples to traditional medicine and health practices and appropriate health services, with sovereign decision making in health programs (35). The intersection many Indigenous peoples experience in their health care journey between pursing traditional health practices in a Westernized health care setting can be negatively impacted by the dismissal of such practices. Many health care professionals may consider traditional medicine and healing to be superstitious practices that hinder evidence-based therapy, cause harm to the patient, and burden the health care system (36). The systemic belief in some health care systems of traditional medicine contributes to denialism by reinforcing the hierarchy of Westernized colonized beliefs of superiority over Indigenous ways of knowing, creating mistrust and lack of autonomy in Indigenous health care experiences (40).

Denialism continues to perpetuate anti-Indigenous racism in health care experiences and inequity for Indigenous peoples. These implications are significant for policy, education, and reconciliation initiatives. Frameworks of denialism need to be challenged to confront the interpersonal, systemic, and institutional conditions which allow anti-indigenous racism to exist. Re-examining and rewriting educational curriculum to include the true history of Canada, presenting Indigenous people as a strong community, able to withstand and address colonial history and portraying media presence appropriately are necessary to combat denialism. Speaking the truth, highlighting the past and amplifying Indigenous knowledge and voices in a sovereign supported manner is mandatory to eliminate denialism. Providing platforms, conducting research in a strength-based approach using Indigenous research methodologies, to clarify how denialist narratives evolve and continue to exist is essential for developing strategies against them.

## 7 Conclusion

The influences and impact of racism continue to exist in healthcare. Colonialist views, concepts of Westernized medicine and stereotypes persist in health care experiences for Indigenous peoples in Canada. To enact change and move toward reconciliation, recognizing and acknowledging the historical events of colonialism by eliminating denialism at all levels is a vital component in addressing anti-Indigenous racism. Illuminating the distinct types of denialism, systemic, interpersonal, and societal, empowers individuals, teams, and health care institutions to acknowledge their collective responsibilities for reconciliation.

## Data Availability

The data that support the findings of this study are available from the corresponding author, Patricia Farrugia, patricia.farrugia@medportal.ca. upon reasonable request.

## Footnotes

1 The term “Indigenous” is used to refer collectively to the original inhabitants of what is now known as Canada, including First Nations People, Inuit, and Métis Peoples. Wherever relevant, the term “First Nations,” “Inuit” and “Métis” will be used when referring to specific Indigenous groups. Following international Indigenous standards, the term “Peoples” is used when referring to Indigenous groups in recognition of their status as governing Nations, and “people” when referring to individuals.
