# Supplementary figures and images for: Denialism: repudiation of anti-Indigenous racism in healthcare in Canada
Source: Front Public Health. 2026 Mar 3;14:1766047. doi: 10.3389/fpubh.2026.1766047 (PMC12992290; doi:10.3389/fpubh.2026.1766047)

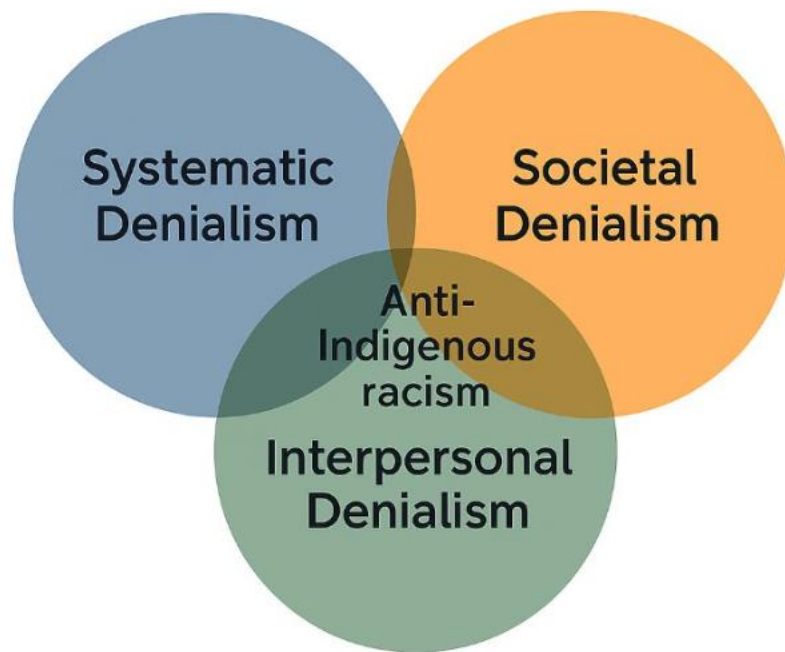

Supplement: Supplementary file 1 [file Data_Sheet_1.PDF]
